# Galangin inhibits epithelial-mesenchymal transition and angiogenesis by downregulating CD44 in glioma

**DOI:** 10.7150/jca.31487

**Published:** 2019-07-25

**Authors:** Daliang Chen, Dengfeng Li, Xiao-bing Xu, Shengcong Qiu, Shi Luo, Erning Qiu, Ziyun Rong, Ji Zhang, Dahai Zheng

**Affiliations:** 1 Department of Neurosurgery, Shunde Hospital, Southern Medical University (The First People,s Hospital of Shunde Foshan), Foshan, China.; 2 Department of Neurosurgery, Shanwei People's Hospital, Shanwei, Guangdong, China.

**Keywords:** galangin, EMT, CD44, suppression, glioblastoma

## Abstract

Galangin (3,5,7‑trihydroxyflavone), a natural flavonoid present in plants, has been reported to possess anticancer properties in various types of cancers comprising glioma. The underlying mechanism, however, has not been fully elucidated. CD44, a hall marker in glioma, has been reported to be associated with epithelial-mesenchymal transition (EMT) and angiogenesis, which play important roles in glioma progression. In this study, we aimed to investigate whether galangin can inhibit EMT, angiogenesis and CD44 expression in glioma. We observed that galangin inhibited the proliferation, migration, invasion and angiogenesis of glioma cells in a dose-dependent manner, suppressed the expression of CD44 and inhibited angiogenesis of glioma cells through downregulating vascular endothelial growth factor (VEGF) in HUVECs. In addition, the overexpression of CD44 in U87 and U251 cells partly abolished the effects of galangin on glioma cells. Moreover, galangin suppressed tumor growth in an intracranial glioma mouse model. These results indicate that galangin is a potential novel drug for glioblastoma treatment due to its ability to suppress of CD44, EMT and angiogenesis.

## Introduction

Malignant gliomas are the most common and lethal brain tumor. Glioblastoma, characterized by rapid growth and high invasiveness, is the most malignant pathological type of glioma 1, 2. Over the past several decades, although a variety of therapeutic approaches including surgery, chemotherapy, radiotherapy and combined modalities, have been developed, the median survival time of newly diagnosed patients with glioblastoma has been less than 15 months 3, 4. Thus, it is urgent to develop new therapeutic agents for glioblastoma.

Accumulating evidence has demonstrated that EMT is related to cancer development and metastasis. The EMT phenotype of cancer cells often refers to the loss of epithelial characteristics and the acquisition of mesenchymal properties, showing strong proliferation, migration and invasion capacities that allow cancer cells to invade adjacent tissues and blood vessels and /or detach from the primary site 5, 6. A typical feature of the EMT process is the variation in mesenchymal markers, such as elevated Vimentin and Snail, which induces the breakdown of cell-to-cell junctions. Though it still remains controversial, emerging evidence on the basis of the neuroepithelial theory confirmed the existence of an EMT-like process in glioblastoma. The activation of the glioblastoma EMT-like program promoted the malignant progression, including migration and invasion *in vitro* and *in vivo*. In addition, therapy targeting EMT has been proven effective 7, 8. EMT can be induced by a variety of molecules and signaling pathways. Among them, CD44 has been demonstrated to play an important role in regulation of EMT 9, 10. Moreover, in recent studies, it was further verified that higher CD44 levels allow the cancer to acquire more malignant abilities, and patients with higher levels of CD44 exhibited a shorter survival time 11-13. Therefore, if CD44 inactivation inhibits EMT, it could prevent the formation and progression of glioblastoma and provide a potential option for glioblastoma treatment.

Angiogenesis, the growth of new capillary blood vessels, is a crucial process for the growth, maintenance and metastasis of solid tumors. Angiogenesis is also a highly complicated process embracing endothelial cell proliferation, migration, invasion and tubular formation. There has been increasing amounts of evidence indicating that the development and metastasis of glioblastoma critically depend on angiogenesis, and the level of tumor angiogenesis has been associated with the prognosis of glioblastoma patients. Based on its importance in tumor progression, Dr. Folkman and collogues proposed the concept of “anti-angiogenic therapy” 14-16. Recently, several studies have verified that the inhibition of tumor angiogenesis could contribute to suppressing the cancer. Angiogenesis could be regulated by multiple molecular and signaling pathways. Convincing evidence has shown that CD44, as a potent angiogenic molecule, contributes to cancer progression 17, 18. Moreover, CD44 plays an important role in angiogenesis by modifying VEGF, which is thought to be an important therapeutic target in glioblastoma 19.

Plants, as sources of phytocompounds that exhibit anticancer properties for a variety of human malignant tumors, have been widely used in folk medicine. Among these phytocompounds, polyphenols are abundant in our daily foods and have received increasing attention in recent years because of their potential benefits in the prevention of diseases such as heart disease and chronic inflammation, as well as reducing the risk at cancer 20, 21. Galangin, 3,5,7-trihydroxyflavone, is a flavonoid that occurs in Alpinia officinarum herbal plants, which are used in Asian as a therapeutic drug. These plants are a rich source of honey, which contains major constituents of propolis, a natural compound secreted by honey bees 22. Galangin exhibits different pharmacological properties including antioxidative, antimutagenic and radical scavenging properties. Studies have reported that galangin presents anticancer effects in different cancer cells, such as hepatocellular carcinoma cells, colon cancer cells, ovarian cancer cells, human mammary tumor cells, melanoma cells, prostate cancer cells and promyelocytic leukemia cells 23-27. Galangin induces apoptosis through the mitochondrial pathway and G0/G1 cell cycle arrest via the reduction of cyclins E, A9 and D310 28. Galangin, at different concentrations, triggers autophagy and apoptosis via the elevation of p53 in HepG2 cell lines 29. TRAIL-induced apoptosis was observed in prostate cancer cells upon treatment with galangin. Though galangin affects cell proliferation and apoptosis in various cancer cells, knowledge on the exact effects and the associated molecular mechanisms of galangin in glioma still remains elusive.

In the present study, we explored the effects of galangin on cell proliferation, migration and invasion in human glioblastoma cell lines. We further investigated whether these effects are due to the regulation of EMT and angiogenesis via the modulation of CD44 expression by galangin in glioblastoma. In addition, we confirmed these findings by overexpressing CD44 in human glioblastoma cells. Furthermore, we examined whether galangin suppresses tumor growth in an intracranial glioma mouse model. Taken together, our findings validate galangin as a potential anticancer agent and provide evidence of CD44 inhibition as an underlying mechanism.

## Methods and Materials

### Chemicals, reagents and antibodies

Galangin was purchased from Sigma-Aldrich (St. Louis, MO, USA). Galangin was dissolved in DMSO and stored at 4°C. Dulbecco's modified Eagle's medium (DMEM) and fetal bovine serum (FBS) were purchased from Gibco (Grand Island, USA). Antibodies against CD44, Snail, Vimentin, ZEB1, VWF, and GAPDH were purchased from Cell Signaling Technology (Beverly, MA). Antibodies against VEGF and Snail were purchased from Abcam (Cambridge, MA).

### Cell culture

The human glioblastoma cell lines U87, U251 and U87-luciferase and HUVECs were purchased from the Chinese Academy of Medical Sciences (Beijing, China). The U87, U251 and U87-luciferase cell lines were cultured in high-glucose Dulbecco's modified Eagle's medium (DMEM) supplemented with 10% fetal bovine serum (FBS), and HUVECs were cultured in endothelial cell medium (ECM) supplemented with 1% endothelial cell growth supplement (ECGS) and 10% fetal bovine serum (FBS). All cells were incubated at 37℃ in a humidified atmosphere in 5% CO2.

### Cell viability assay

Cells were seeded at 4× 10^3^ cells/well in a 96-well plate for 24 hours then treated with paeoniflorin. Subsequently, 10 μl of CCK-8 solution was added to each well, followed by incubation for 1 hour at 37°C. Then, the reaction mixture was measured by the microplate reader.

### Cell invasion assay

The transwell assay of cell invasion was obtained from Corning (Corning, USA). Cells (1×10^5^ in 200 μL DMEM or ECM supplemented with 1% FBS) were seeded in the upper chamber (8 μM) coated with 100 µl of Matrigel (BD Biosciences, CA, USA). The lower chamber was filled with 600 μL of DMEM or ECM supplemented with 20% FBS. After 24 hours, the cells in the lower chamber were fixed by methanol, stained with 0.1% crystal violet in methanol, and photographed in three independent 100× fields for each well. Then, the cells in each field were counted.

### Capillary tube formation assay

HUVECs were cultured at 37 °C in a 24-well plate coated with Matrigel (BD Pharmingen, San Diego, CA). After being treated with the indicated concentrations of paeoniflorin for the indicated time, the formation of capillary-like structures was determined under a light microscope. The number of the formed tubes, which represent the degree of angiogenesis *in vitro*, was scanned and quantitated in five low power fields (100× magnification).

### Chorioallantoic membrane (CAM) assay

Fertilized white Leghorn chicken embryos were randomly divided into two groups with five embryos per group and collected on day 3 in sterile containers for subsequent incubation at a humidified environment at 37.5 °C for 48 hours. On day 5, 20 μM paeoniflorin was then added to a microbial sterilized Whatman filter disk to saturation and placed onto the CAM by breaking a small hole in the superior surface of the egg. PBS was used as a control. On day 7, the CAM was cut, fixed by acetone and viewed under a microscope. Neovascularization around the disk was quantitated by determining the number of angiogenic vessels within the CAM around the disk.

### Reverse transcription polymerase chain reaction (RT-PCR)

Total cellular RNA was extracted using Trizol Reagent (Sigma-Aldrich). Equal amounts of the first-strand cDNAs were synthesized with the FastQuant RT Kit (Tiangen Biotech, Beijing, China). PCRs were performed with Taq DNA polymerase (Takara, Dalian, China). The following primers were used: human CD44: CATCTCAGAACGGTTCATGCC (forward), CATTGTGGGCAAGGTGCTATT (reverse); and human GAPDH: TTGGTATCGTGGAAGGACTCA (forward), TGTCATCATATTTGGCAGGTT (reverse).

### Immunohistochemistry

Tissue slides were incubated for 1 hour at 37°C and deparaffinized. Antigen retrieval was carried out by microwave treatment in citrate buffer for 10 minutes. After peroxidase activity was blocked with 3%H_2_O_2_/methanol for 10 minutes, sections were incubated with normal goat serum for 10 minutes to block nonspecific antibody binding sites. Sections were incubated with the primary antibodies for 1 hour at 25 °C followed by incubation with biotinylated anti-rabbit/mouse IgG and peroxidase-labeled streptavidin for 10 minutes each. Then, the images were acquired by macroscope and analyzed by Image Pro-Plus software.

### Transfection

To induce the overexpression of CD44, cell lines were transfected with plasmids carrying a CD44-flag or a flag only (GeneCopoeia, Maryland Rockville, USA) using Lipofectamine 3000 following the manufacturer's protocol.

### Western blotting

Western blots were performed using cell lysates or xenograft glioblastoma tissue homogenates. Protein was extracted using Pro-prep TM Protein Extraction Solution (iNtRON Biotechnology, Korea) according to manufacturer's instructions. Equal amounts of total protein were separated on 10%-12% sodium dodecyl sulfate-polyacrylamide gel electrophoresis (SDS-PAGE) and transferred to polyvinylidene difluoride membranes (Merck, KGaA, Darmstadt, Germany). The membranes were blocked with 5% BSA at room temperature for 1 h and then incubated with specific primary antibodies overnight at 4 ℃. The appropriate secondary antibodies conjugated with HRP were incubated for 1h at room temperature, and the signal was obtained using Super Signal ECL (Pierce, Rockford, IL, USA).

### Galangin treatment in a U87 xenograft mouse model

Female BALB/c nude mice were obtained from Vital River Laboratories (Beijing, China). Mice were aged 6 weeks and kept under a standard protocol approved by the Institutional Animal Care of Army General Hospital. All procedures involving animals were performed in accordance with the ethical standards of the institution or location in which the studies were conducted. Mice were anesthetized with 3.6% chloral hydrate in 0.9% sterile saline. Each mouse was injected intracranially with 4μl of cultured U87-luciferase cells (5×10^5^ cells per mouse) at a rate of 0.5 μL/min using a Micro 4 Microsyringe Pump Controller (World Precision Instruments, Sarasota, FL) attached to a Hamilton syringe with a 33-gauge needle (Hamilton, Reno, NV) into the mid-right striatum at the following coordinates in mm from bregma: +0.5 anterior-posterior, +2.0 medio-lateral, and -2.8 dorso-ventral. Seven days after cell transplantation, the tumor-bearing mice were distributed into two groups (n = 5 each) and intraperitoneally injected with galangin (200 mg/kg/day) or vehicle. Tumor sizes and body weights were measured once every 9 days. At the end of these experiments, the mice were sacrificed and the tumors were resected and homogenized for Western blotting.

### Bioluminescence imaging

D-luciferin was purchased from Abcam (Cambridge, MA) and resuspended at 100 mg/mL in PBS. For Fluc imaging, mice were injected i.p. with 150 mg/kg body weight of D-luciferin and imaged 10 minutes later using the IVIS^®^ Spectrum optical imaging system fitted with an XGI-8 Gas Anesthesia System (Caliper Life Sciences, Hopkinton, MA). Bioluminescent images were acquired using the autoexposure function. Data analysis for signal intensities and image comparisons were performed using Living Image^®^ software (Caliper Life Sciences).

### Statistical analysis

The data are presented as the mean ± standard deviation of at least three independent experiments. Simple comparisons between two groups were analyzed using independent t-tests. Multiple comparisons between the groups were performed using one-way ANOVA followed by post hoc analysis with LSD or Dunnett's T3 test on SPSS 20.0 software. A P value <0.05 was considered statistically significant.

## Results

### Galangin inhibited proliferation, migration and invasion in glioblastoma

**Figure 1:**
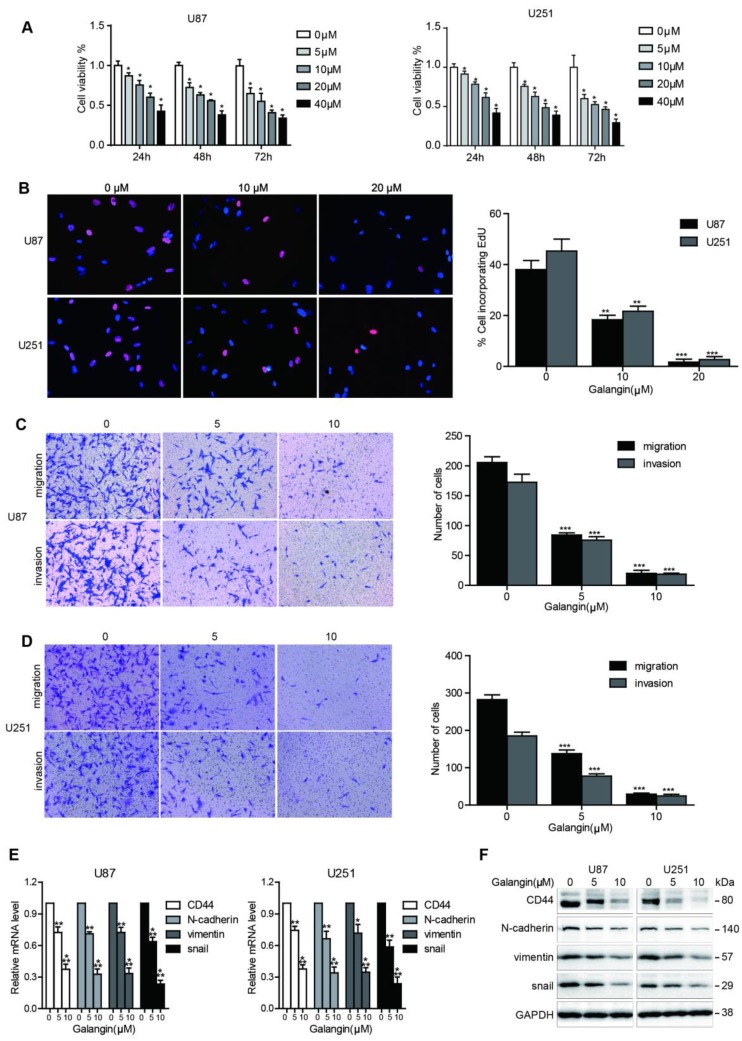
Galangin inhibited cell proliferation, migration, invasion and downregulated CD44 and EMT markers in glioblastoma cells**.** (A) U87 and U251 cells were incubated with different concentrations of galangin for 24, 48 and 72 hours and then cell viability was detected using the CCK-8 assay. (B) U87 and U251 cells were treated for 24 hours with different doses of galangin, then the EdU assay was performed. (C, D) U87(C) and U251(D) cells were treated with the indicated doses of galangin for 24 hours, and transwell assay was applied to examine the migration and invasion. (E, F) U87 and U251 cells were treated with different doses of galangin for 24 hours, and qPCR (E) and Western blotting (F) were applied to analyze the mRNA and protein expression levels of CD44 and the EMT markers. n=3 or 4, and all tests were performed in triplicate. *P<0.05, compared with control (0 μM).

To investigate the effects of galangin on cell proliferation, we performed the CCK-8 assay. As shown in **Figure 1A**, 24, 48 and 72 hours of galangin therapy significantly inhibited cell growth in U87, U251 glioblastoma cell lines in a dose-dependent manner. To confirm the proliferation inhibition effect of galangin on glioblastoma, the EdU assay was used, and as shown in **Figure 1B**, galangin decreased the proliferation rate in U87 and U251 cells in a concentration-dependent manner.

We next conducted a cell migration and invasion assay to evaluate the effects of galangin on glioblastoma cell migration and invasion ability. Low doses of galangin (5 μM and 10 μM) were used in the control and experimental groups to prevent galangin from inducing cell death. Compared with the untreated groups, the galangin-treated group exhibited fewer cells migrating to and invading the bottom of the insert membranes (**Figure 1C-D**). These results demonstrate that galangin significantly inhibited glioblastoma cell migration and invasion.

### Galangin downregulated the expression of CD44 and EMT markers in glioblastoma cell

CD44 has been reported to be an oncoprotein in glioblastoma. Therefore, inhibition of CD44 could be a potentially effective method for glioblastoma treatment. The EMT and angiogenesis processes contribute to glioblastoma progression, and the suppression of the EMT and angiogenesis processes is a promising approach to treat glioblastoma. We next investigated whether galangin can regulate the expression of CD44 and EMT markers. Our results demonstrated that galangin reduced the mRNA and protein levels of CD44, Snail, Vimentin and ZEB1 in a dose-dependent manner (**Figure 1E-F, Supplementary Figure 1**). These results indicate that galangin plays a critical role in the regulation of CD44 and EMT in glioblastoma.

### Overexpression of CD44 abolished the effects of galangin on glioblastoma cells

**Figure 2:**
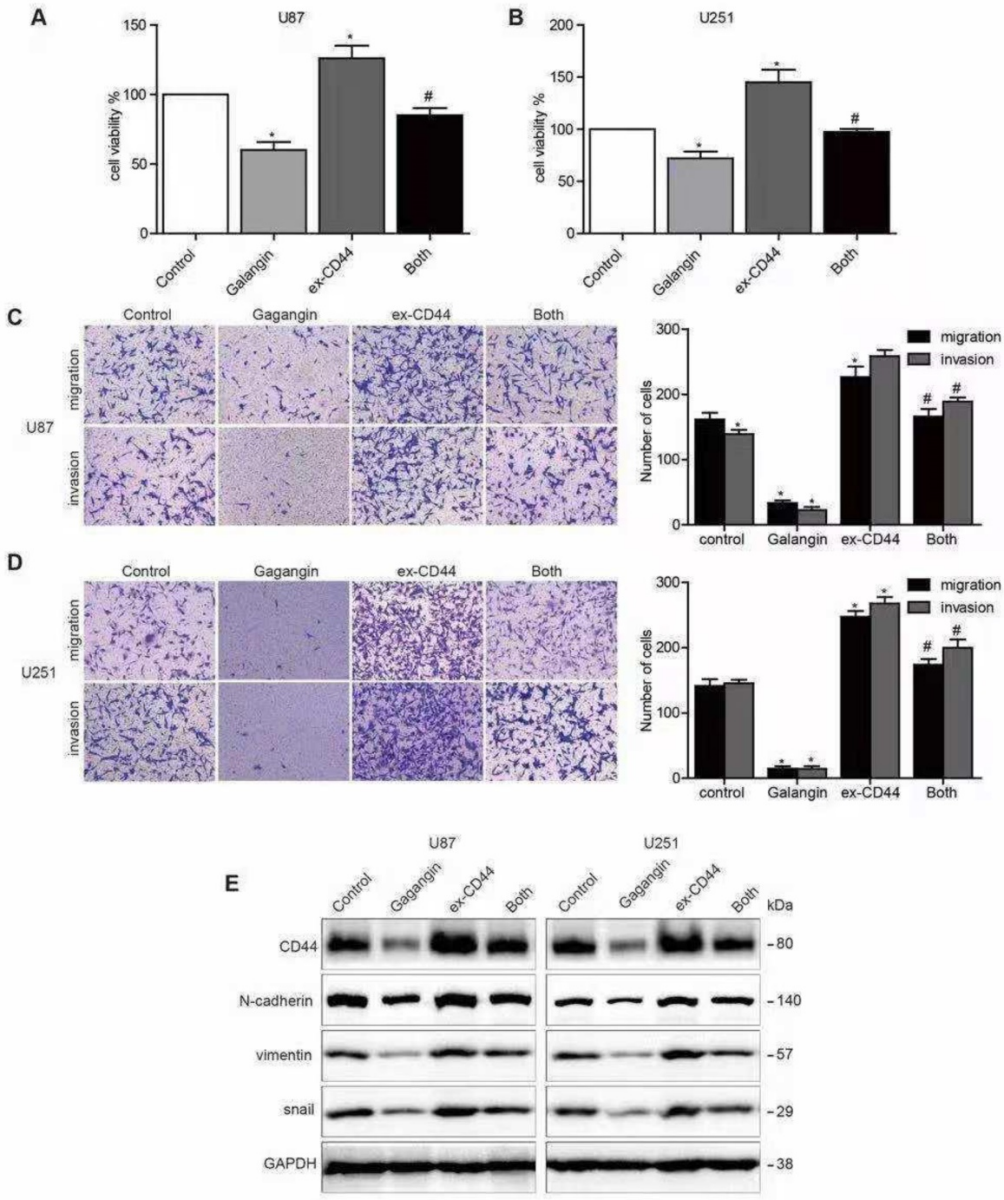
Overexpression of CD44 abolished the galangin-mediated reduction in cell proliferation, migration, invasion and EMT. (A, B) U87 (A) cells and U251 cells (B) transfected with CD44 or vector plasmid for 18 hours were harvested, and 5×10^3^ cells were seeded into a 96-well plate and were incubated with 20 μM galangin or vehicle for 24 hours. A CCK-8 assay was used to examine cell proliferation. (C, D) U87 cells (C) and U251 cells (D) transfected with CD44 or vector plasmid for 18 hours were harvested, and 1×10^4^ cells were seeded into a transwell chamber. Then, a transwell migration and invasion assay was applied to evaluate cell migration and invasion capabilities. (E) U87 cells and U251 cells transfected with CD44 or vector plasmid for 18 hours were then incubated with 10 μM galangin for 24 hours. Western blotting was performed to detect the protein levels. Control: transfection vector; Galangin: transfection vector + μM galangin; ex-CD44: transfection with CD44; Both: transfection with CD44 + μM galangin. *P < 0.05 vs control. ^#^P < 0.05, compared with either galangin treatment or CD44 transfection alone.

To further investigate whether galangin causes anti-EMT effects via the inhibition of CD44 in glioblastoma cells, we generated transfected U87 and U251 cell lines overexpressing CD44 by transfecting the cells with a CD44 cDNA plasmid. Cells with vector transfection were used as CD44-negative controls. The CD44-overexpressing cells were incubated with galangin for 24 hours. We found that overexpression of CD44 increased tumor cell proliferation, and overexpression of CD44 abolished the effects of galangin on proliferation in glioblastoma cells (**Figure 2A**).

We also observed that the upregulation of CD44 enhanced glioblastoma cell migration and invasion. Both glioblastoma cell migration and invasion promoted by the overexpression of CD44 were abolished by treatment with galangin. In addition, we also noticed that the following EMT makers were upregulated after CD44 overexpression: N-cadherin, Vimentin, and Snail. In addition, CD44 overexpression partly abolished the effects of galangin on the downregulation of these markers. These results suggest that galangin plays an anticancer role, at least partly, via the downregulation of CD44-induced EMT in glioblastoma cells.

### Galangin inhibited angiogenesis in HUVECs and the CAM assay and downregulated VEGF

**Figure 3:**
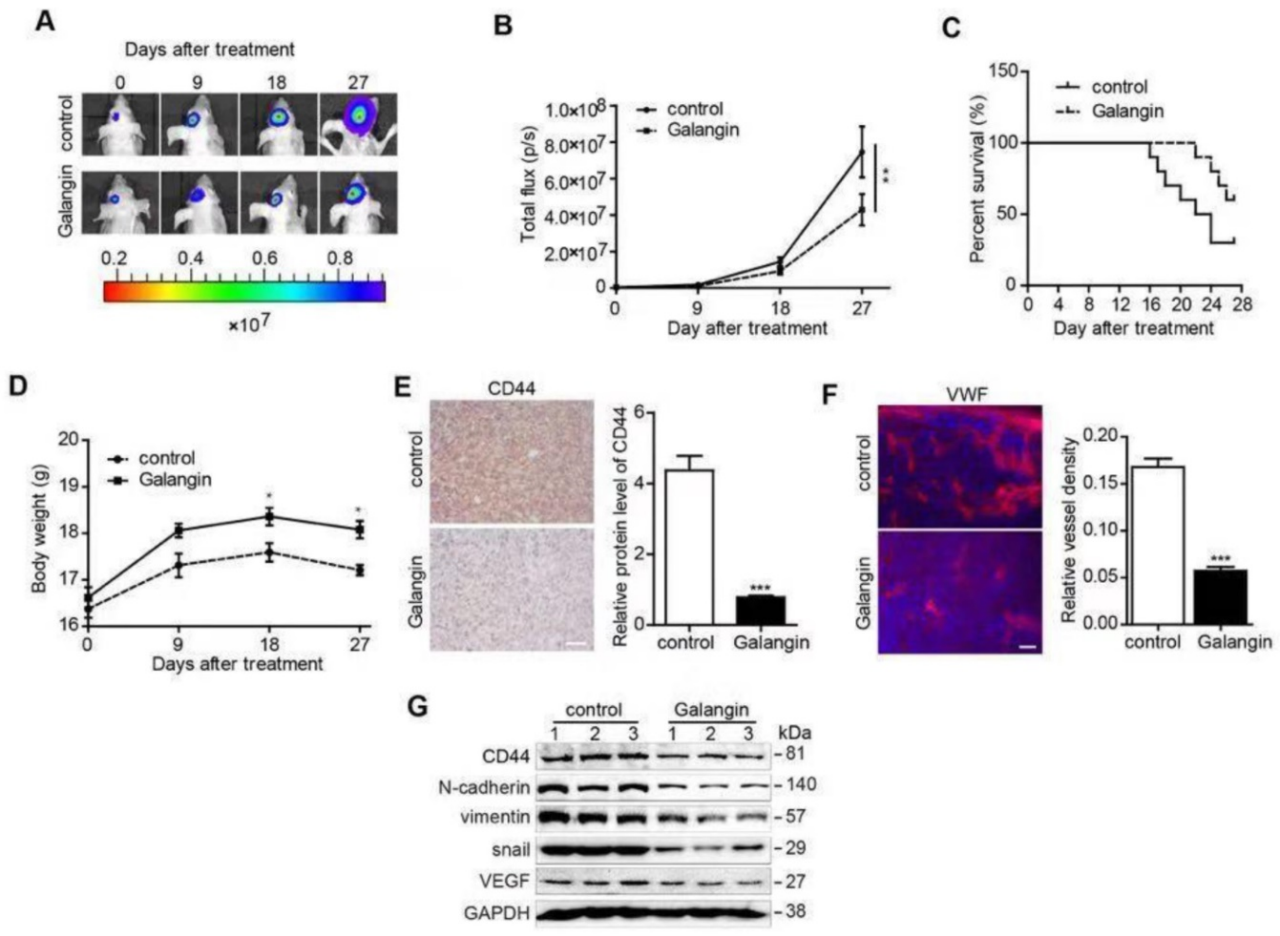
The effect of galangin on angiogenesis in HUVECs. (A-C) HUVECs were treated with the indicated concentration of galangin; then, (A) a CCK-8 assay was used to detect cell proliferation. (B) A tube formation assay was applied to evaluate the angiogenic capability. (C) Western blotting was used to detect the protein level of VEGF. (D) A CAM assay was used to examine the effect of galangin on angiogenesis. CAM were treated with 0 or 20 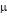
M galangin, and then the number of microvessels were calculated. (E) The overexpression of CD44 abolished the galangin-induced reduction in angiogenesis in HUVECs. HUVECs were transfected with CD44 or vector plasmid for 18 hours and were then incubated with or without 20 μM galangin for 24 hours. The number of tubes was counted, and the protein level of VEGF was detected. Control: transfection vector; Galangin: transfection vector + μM galangin; ex-CD44: transfection with CD44; Both: transfection with CD44 +µM galangin. *P < 0.05 vs control. #P < 0.05, compared with either galangin treatment or CD44 transfection alone.

First, we performed a CCK8 assay to estimate the inhibitory effect on proliferation in HUVECs (**Figure 3A**). Afterwards, low concentrations (5μM and 10μM) of galangin were used to perform the next experiments to exclude the proliferation inhibition effect. To estimate the effect of galangin on tubular formation, we carried out a tube formation assay and found that treatment with galangin significantly suppressed or terminated the formation of vessel-like structures in a concentration-dependent manner (**Figure 3B**). Since galangin could suppress angiogenesis *in vitro*, we performed CAM assays to see if the galangin could repress angiogenesis *in vivo*. A disruption of angiogenesis was observed in galangin-treated chicken embryos, with attenuated microvessels in the CAM and fewer angiogenic vessels compared to the control group (**Figure 3C**). These results suggest that galangin inhibits angiogenesis *in vitro* and *in vivo*. Next, we detected the VEGF protein level after treatment with the indicated concentration of galangin and found that galangin downregulated VEGF in a dose-dependent manner.

### Overexpression of CD44 abolished the effects of galangin on angiogenesis

To further investigate whether galangin causes antiangiogenesis in HUVECs, we generated transfected HUVECs overexpressing CD44 by transfecting HUVECs with a CD44 cDNA plasmid. Cells with vector transfection were used as CD44-negative controls. The CD44-overexpressing cells were incubated with galangin for 24 hours. We found that overexpression of CD44 increased angiogenesis and VEGF protein expression, and overexpression of CD44 abolished the effects of galangin on angiogenesis and VEGF upregulation in HUVECs (**Figure 3E, F**).

### Galangin decreased glioma growth in an orthotopic xenograft mouse model

**Figure 4:**
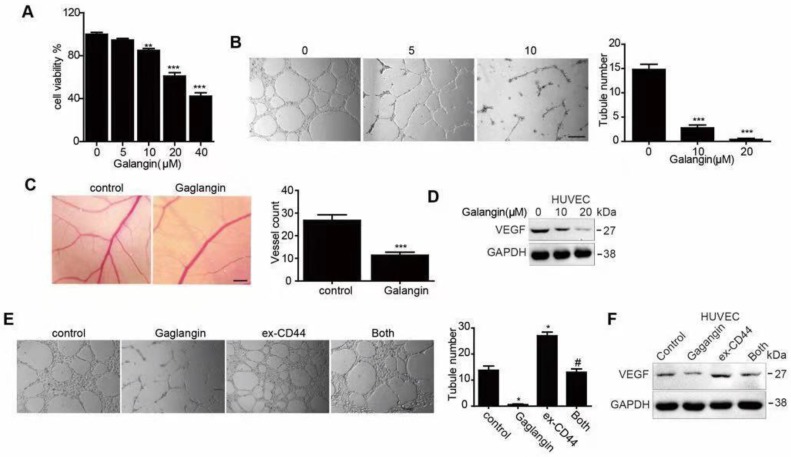
Galangin decreased glioma growth in an orthotopic xenograft mouse model. U87-luciferase cells (5×10^5^) were intracranially injected into the mid-right striatum of 8-week-old male BALB/c nude mice. Seven days after injection, the tumor formation was examined by bioluminescence imaging, and the mice were separated into two groups: those that were intraperitoneally injected with vehicle (control) and those that were intraperitoneally injected with galangin (200 mg/kg/day). Tumor sizes were measured once every 9 days. Bioluminescence imaging was used to measure tumor volume. (A) A representative mouse demonstrating the tumor volume from each group is shown at each time point. (B) The tumor volume of each group was examined at each time point. (C) The survival rate of each group was recorded. (D) The body weight of the mice was measured at the indicated time points. At the end of the experiment or after the mice died, the brains were excised (E) and CD44 (bar, 20 μm) and (F) VWF (bar, 50μm) were stained. The images were analyzed by Image Pro-Plus. (G) At the end of the experiment or after the mice died, tumor tissues were excised from the mice, and protein lysates were performed to estimate the protein expression. *P<0.05, compared with control.

We further explored the inhibitory effects of galangin on glioblastoma in a U87-luciferase orthotopic xenograft mouse model. The results showed that tumor volumes decreased markedly (**Figure 4A, B**), and the survival rate of the tumor-bearing mice was enhanced in the 28-day galangin-treated group compared with that of the control group (**Figure 4C**). In addition, the body weight of the mice in the galangin-treated group exhibited less of a decrease (**Figure 4D**).

Next, we detected CD44 protein levels using the immunohistochemical method and found that CD44 levels were reduced in the galangin-treated group **(Figure 4E**). In addition, we stained the vessel in the tumor tissue from the U87 xenograft mouse brains and found that the vessel density was decreased in the galangin-treated group compared with that in the control group (**Figure 4F**). Furthermore, the endogenous expression of CD44, N-casherin, Vimentin, Snail, and VEGF in tumor tissue from the U87 xenograft mice was suppressed by galangin treatment, indicating that galangin inhibited EMT and angiogenesis in the orthotopic xenograft mouse model **(Figure 4G**). The results of *in vivo* studies are consistent with results of *in vitro* studies, demonstrating that galangin may play a critical role in the suppression of glioblastoma growth in an intracranial tumor model via inhibition of CD44-mediated EMT and angiogenesis.

## Discussion

Emerging evidence has demonstrated that CD44 plays an important role in regulating the EMT and angiogenesis processes, which contribute to tumor initiation and progression. Galangin is an important flavonoid that exhibits a variety of potential effects, such as anti-inflammation and antioxidative stress, through multiple mechanisms 28, 30. It was also reported that galangin plays an inhibitory role in the development of tumors. In the present study, galangin suppressed the proliferation, migration and invasion of glioma cells by inhibiting the EMT process. In addition, we found that galangin could inhibit angiogenesis. We investigated the potential mechanism and found that the effects of galangin on EMT inhibition and angiogenesis suppression involved CD44 downregulation. Finally, galangin suppressed glioma in an intracranial glioblastoma mouse model via the same mechanism that was observed in the *in vitro* experiments.

Natural products appear to be an alternative approach for developing new anticancer agents, and there is an increasing interest in developing new effective drugs for glioma treatment from natural products. Galangin (3,5,7-trihydroxyflavone), a flavonoid found in high concentrations in lesser galangal, displays multiple bioactivities, including anti-inflammation, antioxidative stress and anticancer through a variety of pathways. 28, 30 In a previous study by Mazzio et al, a high throughput screen suggested that galangin could suppress LPS/IFNγ-activated glioma cells 31. In addition, Lei et al reported that galangin could prevent A172 glioma cell invasion by increasing ERK1/2 phosphorylation 32. In our study, we found that galangin could inhibit the EMT process and angiogenesis in U87 and U251 cells, implicating the suppression of CD44.

CD44, a multistructural and multifunctional transmembrane glycoprotein, was initially identified as a receptor that participates in both physiological and pathological processes. CD44 has been reported to be closely associated with the development of various solid cancers. Correspondingly, the inhibition of CD44 has the ability to attenuate the malignant phenotype, slowing cancer progression and reversing therapy resistance. CD44 can affect multiple tumor phenotypes. Molecular studies have revealed that high CD44 expression was related to EMT. In addition, it has been reported that CD44 expression is involved in tumor angiogenesis 9, 33. In the present study, galangin inhibited EMT and angiogenesis in glioma by downregulating CD44 expression.

EMT, as a confirmed mechanism during cancer progression, can promote the migration, invasion, stemness and drug resistance of tumor cells. EMT has emerged as a key regulator of the invasive state. Although the real relevance of EMT in malignant glioma is still controversial, it has been strongly associated with glioma malignancy, and it has been verified that the established groups of EMT-activators in the Twist- and Snail-families enhance glioma cell migration and invasion abilities both *in vitro* and *in vivo*, as displayed in an animal study and in patient-derived specimens 34, 35. Moreover, the mesenchymal subgroup of glioma can acquire the characteristics of the epithelial phenotype. An increasing number of studies have shown that the suppression of the EMT process can inhibit glioma. Furthermore, it has been reported that galangin could suppress the EMT process in renal carcinoma cells. In this study, we verified that galangin can inhibit EMT in glioma.

Angiogenesis, as a necessary condition for sustained tumor growth, plays a critical pathological role in malignant gliomas. In the past few years, numerous studies using bevacizumab (BEV), a humanized monoclonal antibody against VEGF, have been conducted in patients with brain cancers 36. Thus, agents targeting angiogenesis continue to be investigated. Previously, it has been shown that galangin suppressed ovarian cancer cell angiogenesis 37. In addition, in present study, we confirmed the previous results and found an effect of galangin on antiangiogenesis involving the downregulation of CD44.

Though many natural products have shown anticancer effects *in vitro*, only a few among them can play an anticancer role *in vivo*. In the present study, galangin administration displayed an anticancer effect in an *in situ* glioblastoma model, which was consistent with the results obtained from *in vitro* experiments.

In conclusion, we found that galangin acts as a suppressor of EMT and angiogenesis in glioma *in vitro* and *in vivo*. Furthermore, CD44-induced EMT and angiogenesis processes may be the therapeutic targets of galangin in glioma. Galangin may be a potential natural product to treat glioma.

## Supplementary Material

Supplementary figure 1.

## Abbreviations

EMT: epithelial-mesenchymal transition
VEGF: vascular endothelial growth factor
CAM: Chorioallantoic Membrane
