# Supplementary figures and images for: Galangin inhibits epithelial-mesenchymal transition and angiogenesis by downregulating CD44 in glioma
Source: J Cancer. 2019 Jul 25;10(19):4499–508. doi: 10.7150/jca.31487 (PMC6746128; doi:10.7150/jca.31487)

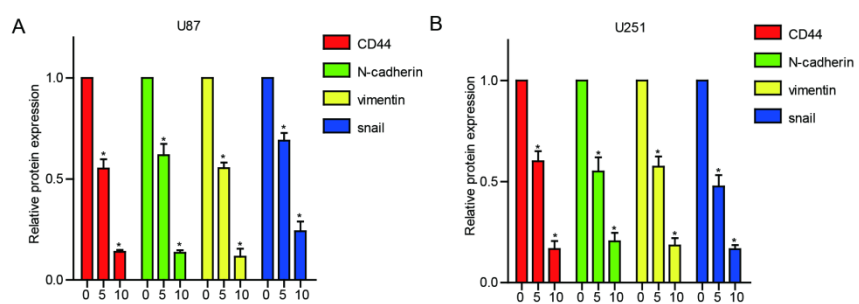

**Supplementary Figure 1.** The quantitative analysis of protein expression for Figure 1F.

Supplement: Supplementary file 1 — Supplementary figure 1. [file jcav10p4499s1.pdf]
